# 

**Published:** 1920-11-06

**Authors:** 


### Matrons' and Sisters' Appointments.

Ballymena Cottage Hospital, Co. Antrim.—Miss Anna B. Logan has been appointed matron. She was trained at the Royal Victoria Hospital, Belfast; she has also been staff nurse at Smiley Hospital, Larne, surgical sister at Belfast Children's Hospital, and has been in charge of the Samaritan Hospital Branch of the Volunteer Force Hospital, Belfast, and served under Queen Alexandra's Imperial Military Nursing Service.

City Maternity Hospital, Wakefield.—Miss Florence Louise Cross has been appointed matron. She was trained at St. Leonard's Infirmary, Shoreditch, and was later maternity and theatre sister and subsequently assistant matron at Southampton Infirmary. From 1911 to 1920 she has been matron of the Wakefield Infirmary.

County Hospital, Ayr.—Miss J. Harley has been appointed theatre sister. She was trained at the Royal Alexandra Infirmary, Paisley, where she was later surgical sister.

County Sanatorium, East Sussex.—Miss Margaret Meldrum has been appointed matron. She was trained at the London Hospital, and has been matron at the Matlock Sanatorium, Mount Vernon Hospital, and the Derbyshire Sanatorium.

Dorchester County Hospital.—Miss Corfield has been appointed sister. She was trained at the Guest Hospital, Dudley, and has since been staff nurse and holiday sister at Bradford Royal Infirmary, and sister of the men's surgical ward at Cameron Hospital, West Hartlepool.

Englethwaite Hospital and Colony (Cumberland County Council).—Miss B. Hennessey has been appointed sister. She was trained at the Stoke-on-Trent Hospital, where she was later staff nurse, and subsequently sister at the Surgical Home, Sutton, Surrey; sister at the Worcester City and County Nursing Institute; sister at the Newcastle Hospital, Staffs; and sister under the Territorial Force Nursing Service.

Royal Hospital for Sick Children, Edinburgh.—Miss Sarah Alice Robinson has been appointed theatre sister. She was trained at the District Infirmary and Children's Hospital, Ashton-under-Lyne, where she was later theatre sister.

Sanatorium for Infectious Diseases, Canton, Cardiff.—Miss Ada Beard has been appointed sister. She was trained at the Greenwich Infirmary and the Isolation Hospital, Cheadle, and was later Queen's nurse at Portsmouth.

Soho Square Hospital for Women.—Miss Dorothy Sellen has been appointed sister. She was trained at the West London Hospital, and has since been staff nurse at the Hospital for Women, Soho Square.

